# Altered GnRH neuron and ovarian innervation characterize reproductive dysfunction linked to the Fragile X messenger ribonucleoprotein (*Fmr1*) gene mutation

**DOI:** 10.3389/fendo.2023.1129534

**Published:** 2023-02-22

**Authors:** Pedro A. Villa, Nancy M. Lainez, Carrie R. Jonak, Sarah C. Berlin, Iryna M. Ethell, Djurdjica Coss

**Affiliations:** Division of Biomedical Sciences, School of Medicine, University of California, Riverside, CA, United States

**Keywords:** Fragile X Syndrome, *FMR1*, GnRH, hypothalamus, FSH, ovary innervation

## Abstract

**Introduction:**

Mutations in the Fragile X Messenger Ribonucleoprotein 1 (*FMR1*) gene cause Fragile X Syndrome, the most common monogenic cause of intellectual disability. Mutations of *FMR1* are also associated with reproductive disorders, such as early cessation of reproductive function in females. While progress has been made in understanding the mechanisms of mental impairment, the causes of reproductive disorders are not clear. FMR1-associated reproductive disorders were studied exclusively from the endocrine perspective, while the *FMR1* role in neurons that control reproduction was not addressed.

**Results:**

Here, we demonstrate that similar to women with *FMR1* mutations, female *Fmr1* null mice stop reproducing early. However, young null females display larger litters, more corpora lutea in the ovaries, increased inhibin, progesterone, testosterone, and gonadotropin hormones in the circulation. Ovariectomy reveals both hypothalamic and ovarian contribution to elevated gonadotropins. Altered mRNA and protein levels of several synaptic molecules in the hypothalamus are identified, indicating reasons for hypothalamic dysregulation. Increased vascularization of corpora lutea, higher sympathetic innervation of growing follicles in the ovaries of *Fmr1* nulls, and higher numbers of synaptic GABA_A_ receptors in GnRH neurons, which are excitatory for GnRH neurons, contribute to increased FSH and LH, respectively. Unmodified and ovariectomized *Fmr1* nulls have increased LH pulse frequency, suggesting that *Fmr1* nulls exhibit hyperactive GnRH neurons, regardless of the ovarian feedback.

**Conclusion:**

These results reveal *Fmr1* function in the regulation of GnRH neuron secretion, and point to the role of GnRH neurons, in addition to the ovarian innervation, in the etiology of *Fmr1*-mediated reproductive disorders.

## Introduction

Mutations in the Fragile X Messenger Ribonucleoprotein 1 (*FMR1*) gene lead to the most common genetic form of intellectual disability and autism, called Fragile X Syndrome (FXS) (1, 2). In addition to intellectual impairment, *FMR1* gene mutations are also associated with reproductive disorders, such as early menopause in females, and macroorchidism in males (3–6). The *FMR1* gene encodes FMR protein (FMRP), an mRNA binding protein that regulates protein levels of its target genes (7, 8). Target mRNAs bound by FMRP encode a variety of proteins, including transcription factors that regulate other genes (9–11). Mutation of this gene that causes FXS entails full expansion of the unstable CGG trinucleotide repeats (>200) that leads to hypermethylation, silencing of the gene and the loss of FMRP. Premutation, in a range of 50-200 repeats, causes Fragile X-associated Tremor/Ataxia Syndrome (FXTAS) and exhibits reduced FMRP levels. While the mechanisms of intellectual impairments following FMRP loss are beginning to emerge, mechanisms of reproductive disorders are not known. Although FMRP is ubiquitous, it is highly abundant in the nervous system. In the brain, FMRP binds mRNAs that encode synaptic proteins, contributing to cognitive dysfunctions in FXS (9–11). The effect of *FMR1* mutations on the cortex and hippocampus have been analyzed (12, 13), however, how mutations affect hypothalamic functions have not been examined. Herein, we investigated the effects of FMRP loss in reproduction, specifically on a population of hypothalamic neurons that regulate the hypothalamus-pituitary-gonadal axis.

Given that *FMR1* gene mutations are also associated with reproductive disorders (3–6), combined with increasing infertility rates (14, 15), it is critical to examine *FMR1* role in the reproductive axis. Reproduction is controlled by gonadotropin-releasing hormone (GnRH)-secreting neurons from the hypothalamus (16, 17). GnRH is secreted in a pulsatile fashion into the hypophysial-portal system, to regulate synthesis and secretion of pituitary gonadotropin hormones, luteinizing hormone (LH) and follicle-stimulating hormone (FSH), which in turn regulate gonadal function (18). Synchronization of GnRH secretion is determined by an upstream regulatory network. One population of such afferent neurons are GABAergic neurons from the mediobasal hypothalamus (19–25). Although GABA is the primary inhibitory neurotransmitter in the brain, GABA is excitatory for GnRH neurons (20, 26–28). Changes in GnRH neuron connectivity and its innervation control neuropeptide pulsatile secretion and consequently gonadotropin hormone levels.

Contrary to the hypothalamus, gonadal roles of FMRP have been analyzed in several reports. Macroorchidism in men affected with FXS (5) is caused by increased Sertoli cell proliferation during development (29). In women, mutations in the *FMR1* gene comprise the largest number of cases with the known genetic causes of early cessation of reproductive function (4, 30, 31). *FMR1* exhibits X-linked dominant inheritance pattern and associated disorders have a high penetrance (32), which contributes to their high incidence (33). Premature ovarian failure is an infertility disorder affecting 1% of reproductive age women that lose ovarian function before the age of 40 (34, 35). Women with early menopause experience not only early infertility, but increased risk of cardiovascular disease, osteoporosis and depression (36). Premutations of the *FMR1* gene are primarily associated with the early loss of reproductive function, which may be due to the much higher prevalence of premutations compared to full mutations. Although to a lesser degree, premutation also causes lower levels of FMRP, and thus, lack of FMRP may cause pathologies in both premutation and full mutation (37). Contrary to men, premature ovarian failure in women with *FMR1* mutations is not a developmental disorder. Previous studies in mice (31) and humans (6, 38–40), did not find differences in primordial follicle numbers, indicating that early follicular development is not affected. Women affected with mutations have elevated FSH (40, 41), which stimulates cyclic recruitment of a wave of ovarian follicles into the growing pool in each menstrual or estrous cycle (42). However, it is still not clear how *FRM1* mutations lead to early infertility.

With a high prevalence of *FMR1* mutations in women of child-bearing age, investigating FMRP-mediated effects on reproductive function will help us understand the mechanism of early menopause. Previous studies that examined ovarian hormone levels or follicle development, failed to explain early infertility. In this study, we first examined the reproductive function in female *Fmr1* KO mice and determined that the mouse model lacking the *Fmr1* gene mimics the reproductive deficits observed in women with *FMR1* mutations associated with reduced FMRP levels. We further observed that *Fmr1* KO mice had larger litters and more corpora lutea in the ovaries, but normal primordial follicle count, indicating increased recruitment. In addition, we found an increase in gonadotropin levels, inhibin B and steroid hormone levels, which indicated that ovarian feedback is present. We further analyzed ovarian vascularization and innervation, and observed changes that may explain increased FSH. Ovariectomy however, revealed a hypothalamic contribution to increased LH. We then analyzed alteration in gene expression and protein levels of critical hypothalamic molecules, and examined the consequence of FMRP loss on hypothalamic GnRH neurons, that have been neglected for their potential role in the etiology of early menopause, and show changes in GABAergic innervation. We show higher LH pulse frequency, before and after ovariectomy, which indicates higher GnRH neuron activity, correlating with alterations in GnRH neuron connectivity. Therefore, central mechanisms, most likely *via* alterations in the activity of GnRH neurons, in addition to ovarian effects, can contribute to increased gonadotropins and higher recruitment of follicles leading to reproductive dysfunction in women with *FMR1* mutations.

## Materials and methods

### Animals

All animal procedures were performed with the approval from the University of California (Riverside, CA) Animal Care and Use Committee and in accordance with the National Institutes of Health Animal care and Use Guidelines. Breeding pairs of FVB.129P2-Fmr1tm1Cgr*/J* (*Fmr1* KO) and their congenic controls (WT) mice were obtained from Jackson Laboratories and bred in-house. Mice were maintained under a 12-h light, 12-h dark cycle and received food and water *ad libitum*. Since we were interested in determining the mechanisms of premature ovarian failure in women with mutations in the *FMR1 gene*, only female mice were used for our studies. Estrous cycle stage was determined with vaginal smears and females were collected in a specific estrous cycle stage, as indicated for each analysis. For fertility studies, *Fmr1* KO females and WT controls were housed with WT males and their litters and numbers of pups per litter were recorded. Ovariectomy was performed, as described before, using 8-week-old mice (43). Animals were allowed to recover and seven days later blood was collected for hormone analyses as described below.

### Hormone assay

Previous studies using FXS mouse models demonstrated that these mice have heightened response to stress and altered levels of glucocorticoids (44), which can lead to a decrease in luteinizing hormone (LH) levels (45, 46). To reduce stress, animals were acclimated by daily handling and tail massage for two weeks prior to hormone measurements. For LH measurements, blood was collected from the tail and analyzed by an in house ultra-sensitive ELISA. The capture monoclonal antibody (anti-bovine LH beta subunit, 518B7) was provided by Janet Roser, University of California. The detection polyclonal antibody (rabbit LH antiserum, AFP240580Rb) was provided by the National Hormone and Peptide Program (NHPP). HRP-conjugated polyclonal antibody (goat anti-rabbit) was purchased from DakoCytomation (Glostrup, Denmark; D048701-2). Mouse LH reference prep (AFP5306A; NHPP) was used as the assay standard. Assay sensitivity is 0.016 ng/ml, while intra-assay coefficient of variation is 2.2% and inter-assay coefficient of variation was 7.3% at the low end of the curve. Other hormone assays were performed by the University of Virginia, Ligand Core using serum that was obtained from the inferior *vena cava* and serum prepared per their instructions. The University of Virginia Center for Research in Reproduction Ligand Assay and Analysis Core is supported by the Eunice Kennedy Shriver NICHD/NIH Grant R24HD102061. FSH was assayed by RIA using reagents provided by Dr. A.F. Parlow and the National Hormone and Peptide Program, as previously described (47). Mouse FSH reference prep AFP5308D was used for assay standards. Inhibin and steroid hormone levels were analyzed using validated commercially available assays, information for which can be found on the core’s website: http://www.medicine.virginia.edu/research/institutes-and-programs/crr/lab-facilities/assay-methods-page and reported in (48). Limits of detection were 2.4 ng/ml for FSH, 300 pg/ml for progesterone, and 100 pg/ml for testosterone. Intra- and inter-assay coefficients of variation were 6.9%/7.5%, 6.0%/11.4% and 4.4%/6.4% for FSH, progesterone (P) and testosterone (T), respectively. Each animal used for each hormone analysis is represented as a dot in the figure.

### Pulsatile LH levels

Pulsatile secretion of LH strictly corresponds to GnRH secretion (49, 50). LH is used as an indicator of GnRH secretion, since GnRH secretion into median eminence cannot be measured in mice. To ascertain if LH or GnRH neuron secretion is affected, we measured LH pulses and used an ultrasensitive ELISA assay for LH that allows for LH measurement in 5 µL of whole blood (51). Mice were acclimated for 2 weeks by daily tail massage. 10µL of blood was collected every 8 min for 3 hours from the tail vain (45, 52). LH levels were analyzed using ELISA described above. LH amplitude was determined by subtracting the LH value at the peak from the basal value prior to the onset of the pulse and averaged for each mouse. Mean LH concentration was calculated by averaging LH values, while pulse frequency was determined using freeware DynPeak algorithm (53).

### Nanostring analysis of hypothalamic gene expression

8-week old female mice in diestrus were perfused with ice cold PBS, brains rapidly removed and flash frozen in isopentane on dry ice. Coronal brain sections of 500 μm were obtained using vibratome, hypothalamus dissected, RNA isolated using the RNAqueous^®^-Micro Kit (Ambion) and quantified using Nanodrop. Gene expression in 50 ng RNA per sample was analyzed using the Nanostring instrument as described before (54), according to manufacturer’s instruction, with the nCounter Mouse Neuroinflammation Panel (770 genes, gene list available at the manufacturer’s website). The panel was customized with an addition of 30 custom probes for: *Gnrh1, Kiss1, Kiss1r, Pdyn, Tac2, Tac3r, Agrp, Npy, Pomc, Cart, Mc3r, Mc4r, Hcrt, Ghrh, Crh, Trh, Oxt, Avp, Prl, Prlr, Adcyap1, Slc6a3, Slc32a1, Slc17a6, Th, Lif, Lifr, Gabra5, Gabra1, Gabrg2*. Only samples with an RNA integrity number RIN over 7 were used after passing QC, with no imaging, binding, positive control, or CodeSet content normalization flags. Data analysis was performed using nSolver Analysis Software 4.0, including nCounter Advanced Analysis (version 2.0.115). Genes with the expression lower than the limit of detection after background subtraction, and compared to negative controls included in the panel, were excluded. Seven housekeeping control genes that are included in the panel, were used for normalization. A heatmap of differentially expressed genes (DEG) was created using Heatmapper software from University of Alberta (Edmonton, Canada (55);). Results are plotted in the Volcano plot as log fold change *vs*. log p-value, and genes with changes higher than 20% were indicated with colors in the figures: red indicates genes higher in KO compared to WT, while green indicates genes that are higher in the WT compared to KO. Genes with significant changes in expression are indicated above the dashed line. Gene ontology (GO) enrichment analysis of the DEG genes was performed using the ShinyGo 0.76.3 platform (South Dakota State University (56)). False discovery rate (FDR) cutoff was 0.05, with the pathway minimum set to 10. Data is deposited in GEO repository (accession number GSE222723).

### qPCR analysis

Total RNA was extracted from ovaries using Trizol (Invitrogen, CA), and from hypothalamus and pituitary using the RNAqueous^®^-Micro Kit (Ambion), quantified using Nanodrop and the same amount per sample reverse transcribed using Superscript IV (Invitrogen, CA). To dissect the hypothalamus, coronal brain sections of 300 μm were obtained using vibratome. Sections containing anterior hypothalamus and posterior hypothalamus were used to dissect the 1 mm x 1 mm mediobasal portion for RNA isolation. Conditions and primers were reported before (57–61).

### Histological analyses and immunohistochemistry

WT controls and *Fmr1* KO mice were anesthetized, perfused with 20 ml PBS and 20 ml 4% paraformaldehyde; and tissues were collected. Ovaries were fixed in 4% paraformaldehyde, embedded in paraffin, and cut to 20 μm sections. Slides were deparaffinized in xylene, rehydrated and H&E stain was performed to count ovarian follicles. For ovarian vasculature and innervation studies, frozen floating sections were stained with antibody to CD31 (1:2000 dilution, 553370, BD Biosciences) or with antibody to tyrosine hydroxylase (TH, 1:5000, ab112, Abcam) for 48 hours at 4°C, followed by overnight incubation with goat anti-rat IgG-Alexa 488 (1:2000, A11006, Vector Laboratories, Burlingame, CA) or goat anti-rabbit IgG-Alexa 488 (1:1000, A11034, Vector Laboratories, Burlingame, CA), respectively. Vascularization of corpora lutea and antral follicles was quantified by the mean fluorescent intensity (MFI) using Fiji ImageJ. Ovarian innervation was quantified by counting the number of neuronal projections in direct contact with follicles or corpora lutea.

Hypothalami were sectioned to 100 μm sections. Sections containing organum vasculosum laminae terminalis (OVLT) where GnRH neurons are located, were blocked and stained for GnRH using rabbit anti-GnRH antibodies (1:10,000 dilution) kindly provided by Greg Anderson (University of Otago; Dunedin, New Zealand (62)), GABAγ2 receptor subunit (1:10,000 dilution, guinea pig anti-GABAγ2, Synaptic systems 224 004), VGAT (1:5,000, mouse anti-VGAT, Synaptic systems 131 011) for 72 hours at 4°C. After PBST washes, slides were incubated overnight at 4°C with secondary antibodies goat anti-rabbit IgG-Alexa 488 (1:1000, A11034, Vector Laboratories, Burlingame, CA); anti-mouse IgG-Alexa 594 (1:1000, A11032, Vector Laboratories, Burlingame, CA); anti-guinea pig–biotin (1:1000, BA-7000) followed by streptavidin-Cy5 (1:1000, 434316, Vector Laboratories, Burlingame, CA). Secondary antibody-only controls were performed to determine antibody specificity. To determine puncta density, we followed our established protocol as previously published (60, 63–66). Puncta were counted in the individual neurons, by an investigator blinded to the group, where at least 45 μm of the axon proximal to soma can be observed using z-stack acquired by confocal Leica SP2 microscope. At least 15-20 individual neurons from 4-5 different sets of mice were counted. 3–D reconstruction was performed by Imaris software (Bitplane, Inc; Concord, MA).

Immunostaining for FMRP was performed using antigen retrieval methods, as previously described (67). Slices were stained overnight with mouse anti-FMRP (1:1000; Developmental Studies Hybridoma Bank, catalog #2F5-1-s, RRID: AB_10805421). Secondary antibody was donkey anti-mouse Alexa 594 (1:300; Molecular Probes, A-21202). Slices were mounted on slides with Vectashield mounting medium containing DAPI (Vector Laboratories, H-1200).

### Western blot

Whole cell lysates were obtained from the dissected hypothalami from WT controls and *Fmr1* knockout mice. The same amount of protein from each sample, determined by Bradford assay, was resolved on SDS-PAGE, transferred on nitrocellulose membrane and probed for: GABAγ2 receptor subunit (1:1000, 14104-1-AP, Proteintech), NMDAR1 (1:1000, 32-0500 Invitrogen), NMDAR2B (1:1000, 07-632 EMD Millipore), postsynaptic density protein 95 (PSD-95; 1:1000, 3409, Cell Signaling), microtubule-associated protein 2 (MAP2; 1:5000, ab5392, Abcam) or β-tubulin (1:1000, sc-9104, Santa Cruz Biotechnology). Bands were quantified using ChemiDoc imaging system (Bio-Rad, Hercules, CA).

### Statistical analyses

Statistical differences between WT control and *Fmr1* KO mice (p < 0.05) were determined by t-test, or ANOVA when appropriate, followed by Tukey’s *post-hoc* test for multiple comparisons using Prism software (GraphPad, CA).

## Results

### *Fmr1* knockout female mice experience early cessation of reproductive function

**Figure 1:**
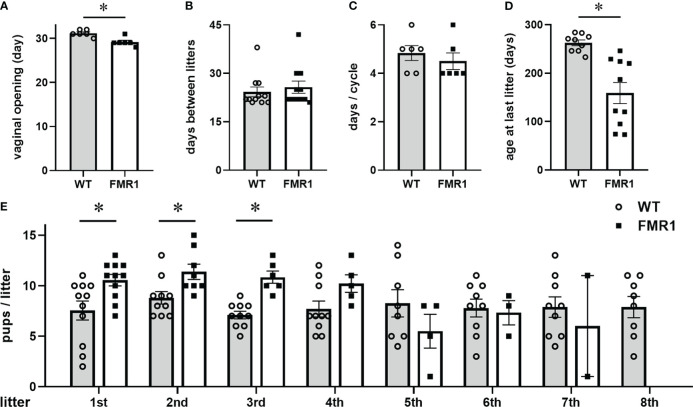
*Fmr1* knockout (KO) females stop reproducing early. **(A)** *Fmr1* KO females (FMR1, white bars represent group mean +/- standard error, and each black square represents one animal) experience early vaginal opening, an external sign of puberty, at postnatal day (p) 29, compared to wild type controls at p31 (WT, gray bars represent mean +/- standard error, open circles represent each animal); **(B)** *Fmr1* KO females have litters at the same rate as WT controls; **(C)** No difference in the length of the estrous cycle; **(D)** Determined by the age at the last litter, *Fmr1* KO females stop reproducing early at p163, compared to WT controls at p263; Each point represents one animal, and bars represent group means +/- standard error. Statistical significance (*, p < 0.05) was determined with t-test followed by Tukey’s *post hoc* test. **(E)** *Fmr1* KO females have more pups per litter in the first three litters. Each circle represents one litter produced by WT females, while each square represents one litter produced by *Fmr1* KO females, with number of pups in 1^st^ – 8^th^ litter indicated. The number of *Fmr1* KO females that continue to produce litters, indicated by squares, decreases gradually. Bars represent group means +/- standard error, * indicates statistically significant difference between WT and KO.

Since women with *FMR1* mutation experience increased risk of early menopause, to begin investigating the role of FMRP in reproductive function, we first determined if the lack of FMRP in female *Fmr1* KO mice can mimic reproductive dysfunctions observed in women with reduced levels of FMRP due to *FMR1* mutation. In affected people, full expansion of the unstable CGG trinucleotide repeats (>200) leads to the loss of FMRP and FXS. CGG expansion in a range of 50-200 repeats, or *FMR1* premutation, exhibits reduced FMRP levels and FXTAS. Due to differential methylation between human and mouse genes, the *Fmr1* KO is a widely used mouse model to study Fragile X Syndrome and is considered a better model than putative mimics of the CGG repeat expansion (68, 69). Our study showed that *Fmr1* KO female mice experienced early vaginal opening, an external sign of puberty in mice (**Figure 1A**, FMR1, *Fmr1* knock-out (KO); WT, wild-type control; each point represents a mouse, while bars represent group average). *Fmr1* KO mice demonstrated vaginal opening at postnatal day 29 (p29), compared to p31 in WT controls. At 8 weeks of age, we paired *Fmr1* KO females and control WT females, with control males and recorded birth dates and number of litters, and the number of pups in each litter, until they stopped reproducing. There was no difference in the length of time between litters (**Figure 1B**) or in the estrous cycle duration (**Figure 1C**). We determined that *Fmr1* KO female mice exhibited early cessation of reproductive function, which is similar to early menopause in women with *FMR1* mutation or premutation (**Figure 1D**, each point represents a mouse) (70, 71). *Fmr1* KO mice stopped having litters at 5.5 months of age and an average age of the last litter was p163 (FMR1, black squares), compared to p263 for WT control females (WT, open circles). Similar to the penetrance in women with mutations in the *FMR1 gene* (32), we observed different degrees of premature cessation of reproductive function. *Fmr1* KO females split into two groups based on the age at last litter: females more severely affected with the mutation that stopped reproducing at p97, and less severely affected females that had the last litter at p221. Nonetheless, the difference between controls (p263) and less affected females (p221) was also statistically significant. We counted the number of pups in the litters and determined that *Fmr1* KO females had larger first three litters (**Figure 1E**). The average litter size of the first litter was 10.6 pups for *Fmr1* KO and 7.5 pups for controls; the average size of the second litter was 11.4 pups for *Fmr1* KO and 8.8 pups for controls, and the average size of the third litter was 10.8 pups for *Fmr1* KO and 7.1 pups for controls. However, while control females continued to produce litters for at least 10 litters, the number of *Fmr1* KO females that continued to produce litters decreased after each litter, and only 5 out of 11 *Fmr1* KO females had a fourth litter, while none had eighth litter. There was no difference in weight at any age between KO and control females and thus, change in reproductive function does not stem from a difference in weight. Therefore, similar to women with a mutation in the *FMR1 gene*, *Fmr1* KO female mice experience early cessation of reproductive function.

**Figure 2:**
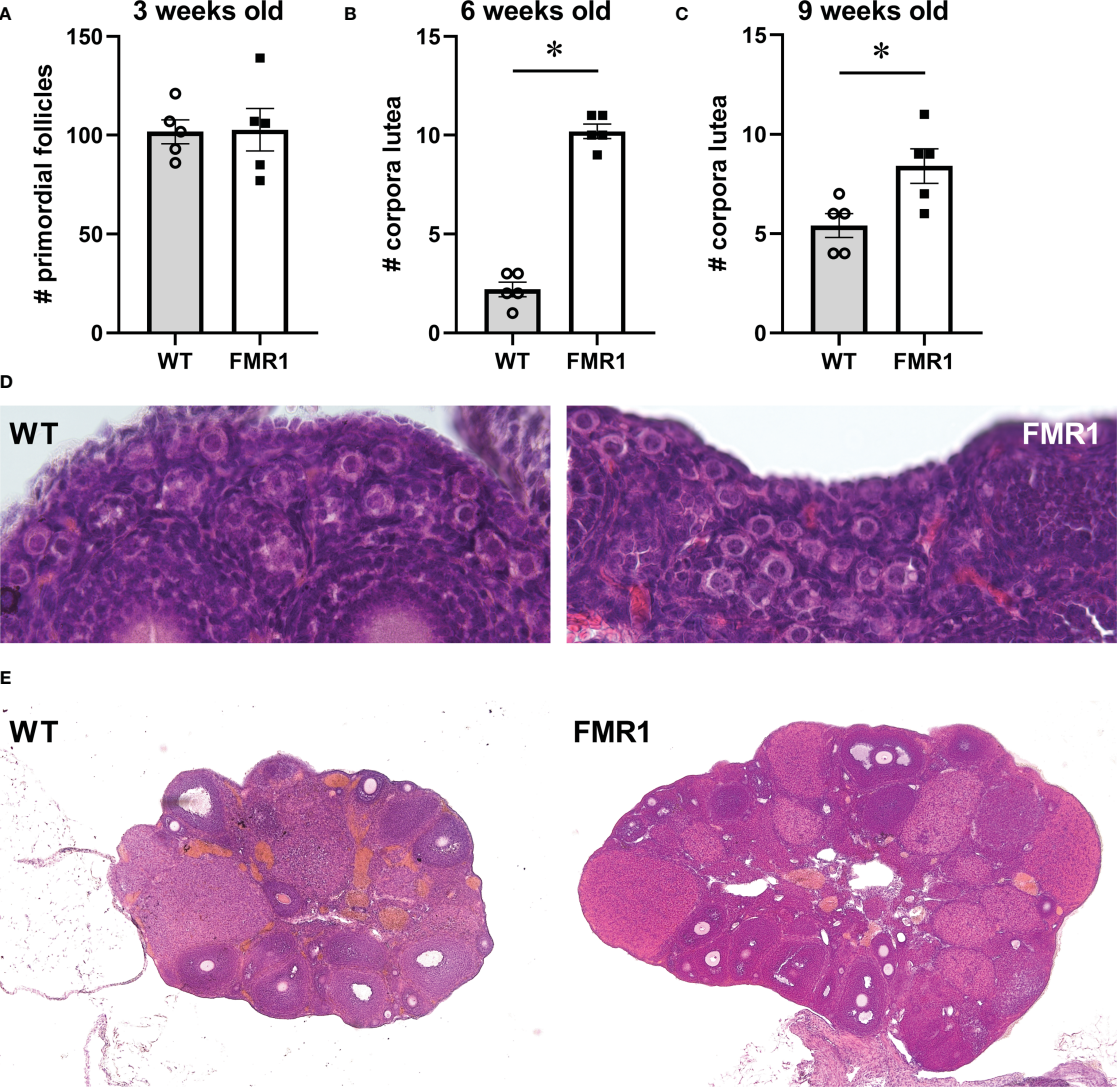
Ovarian histology demonstrates more corpora lutea in young *Fmr1* KO mice. *Fmr1* KO females (FMR1, white bars represent group mean +/- standard error while each black square represents one animal) were compared to wild type controls (WT, gray bars represent mean +/- standard error, each open circle represents one animal). **(A)** Primordial follicles were counted at 3 weeks of age. Each point represents one mouse, and an average of 4 separate 1x10^-8^ m^2^ areas in the ovary cortex of each mouse. **(B, C)** corpora lutea were counted at 6 **(B)** and 9 weeks of age **(C)** throughout each ovary. **(D)** representative images of ovaries from 3-week old mice to observe primordial follicles (630x). **(E)** representative images of ovaries at 6 weeks of age to observe numbers of corpora lutea (40x). Statistical significance, indicated with * (p < 0.05) was determined with t-test followed by Tukey’s *post hoc* test.

To examine if early cessation of reproductive function is due to initially diminished ovarian reserve or to an accelerated loss of follicles, we counted the number of primordial follicles in pre–pubertal females at 3 weeks of age (p21, **Figure 2A** count, and **Figure 2D** representative images). Three equal size areas of the ovarian cortex were selected per mouse, primordial follicles counted, and the numbers were averaged for each mouse. There was no difference in the number of primordial follicles between *Fmr1* KO and WT females, indicating that development of the initial pool is not affected by the *Fmr1* loss. The analysis of the number of corpora lutea (CL) in the ovaries at 6 weeks of age (p42) showed that *Fmr1* KO females had over 4 times more corpora lutea than WT controls. *Fmr1* KO had 10.2 average number of corpora lutea per ovary compared to 2.2 corpora lutea per ovary in controls (**Figure 2B** count, **Figure 2E** representative images). Given that *Fmr1* KO females exhibited early vaginal opening, to confirm that the increase in corpora lutea in KO females does not stem from early puberty, we counted the number of corpora lutea at 9 weeks of age (at p63; **Figure 2C**). At p63, an average number of corpora lutea per ovary was significantly higher in *Fmr1* KO females (8.6 corpora lutea) compared to control WT mice (5.4 corpora lutea). Together, these results demonstrate that *Fmr1* KO female mice stop reproducing early, have the same primordial follicle pool, but increased number of corpora lutea corresponding to the larger litter size in young animals, indicating potentially higher recruitment to the growing pool in young animals.

### *Fmr1* KO females exhibit increased gonadotropin and ovarian hormone levels

**Figure 3:**
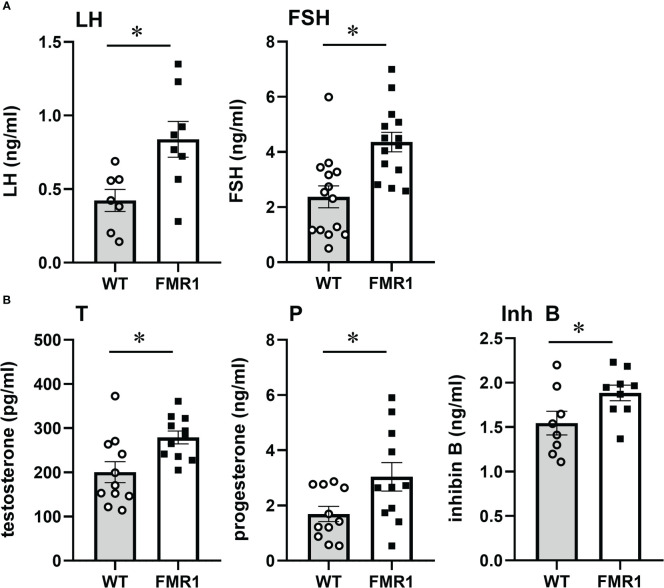
*Fmr1* KO mice have higher LH and FSH. Serum levels of LH and FSH, **(A)** testosterone (T), progesterone (P) and inhibin B (Inh B) **(B)** in diestrus females in WT controls and *Fmr1* KO. Each point represents one animal, while bars represent group means +/- standard error. LH was sampled from the tail tip after acclimatization to handling, to minimize stress and prevent exposure to isoflurane that may affect LH levels. FSH, T, P, Inh B samples are obtained from inferior vena cava. Statistical significance, indicated with a * (p < 0.05) was determined with t-test followed by Tukey’s *post hoc* test.

Given that ovarian function is regulated by gonadotropin hormones from the pituitary, we analyzed the hormone levels in female mice in diestrus at 9 weeks of age (**Figure 3**). LH and FSH levels were significantly higher in diestrus *Fmr1* KO. LH doubled in KO to 0.84 ng/ml from 0.42 ng/ml in controls. Serum FSH was also higher with 4.2 ng/ml in KO, compared to 2.3 ng/ml in diestrus controls (**Figure 3A**). These results may demonstrate that high FSH leads to higher recruitment of follicles in the growing pool, which together with high LH results in more corpora lutea. Since LH and FSH β-subunit transcription, that is unique for each hormone, precedes changes in hormone concentration in the circulation, and fluctuations in mRNA levels in the gonadotrope correlate with concentration of the hormones (18), we analyzed pituitary mRNA levels (**Supplemental Figure 1**). Both *Lhb* (LHβ) and *Fshb* (FSHβ) expression was increased in *Fmr1* KO mice, while expression of the common *Cga* (αGSU, Glycoprotein hormones common subunit alpha), *Gnrhr* (GnRH receptor) or other pituitary hormones was unchanged. These results indicate that concentrations of LH and FSH in the circulation correlate with β-subunit mRNA levels.

Previous studies postulated that ovarian impairment contributed to diminished negative feedback, which in turn caused increased FSH observed in affected women (40, 41). To address this possibility, we analyzed ovarian hormones that provide feedback to the hypothalamus and pituitary in 8-week-old diestrus females before cessation of reproductive function. Steroid hormones primarily provide feedback to the hypothalamus, while inhibin regulates FSH levels (72–77). Testosterone was significantly increased in *Fmr1* KO female mice, 279 pg/ml in KO compared to 200 pg/ml in controls (**Figure 3B**, T). Progesterone was elevated as well to 3 ng/ml in *Fmr1* KO from 1.7 ng/ml in controls (**Figure 3B**, P), demonstrating that negative feedback is present and increased LH cannot be explained by reduced negative feedback. We also analyzed inhibin B levels in diestrus females in the circulation, and determined that inhibin B was higher in KO mice, 1.9 ng/ml compared to 1.5 ng/ml in controls (**Figure 3B**, Inh B). Our results demonstrate that inhibin B is higher in young animals, which may be a result of larger number of follicles, or alternatively that is stimulated by higher FSH levels. This means that inhibin feedback is also present, and cannot explain elevated FSH. This implicates central mechanisms, rather than ovarian insufficiency in the reproductive phenotype of *Fmr1* KO mice. Together, our results demonstrate elevated gonadotropin hormone levels, higher testosterone and progesterone, more corpora lutea, larger litters in young animals, and early cessation of reproductive function.

### Ovariectomy reveals hypothalamic and ovarian contribution to endocrine changes

**Figure 4:**
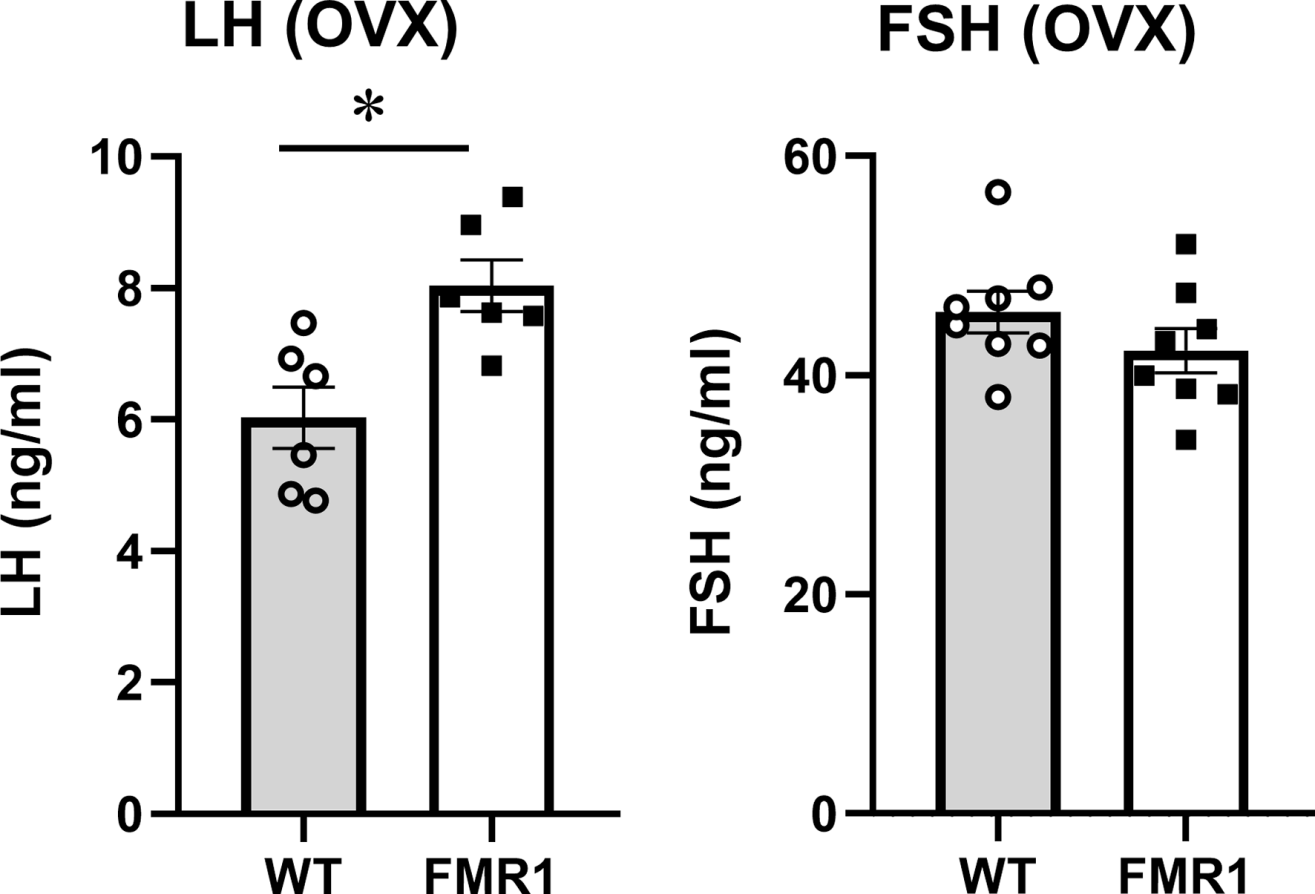
Ovariectomized *Fmr1* KO mice have higher LH. One week after ovariectomy, LH and FSH samples were collected as in **Figure 3**. Each point represents one animal, while bars represent group means +/- standard error. Statistical significance, indicated with a * (p < 0.05) was determined with t-test followed by Tukey’s *post hoc* test.

To discern ovarian contribution from the hypothalamic origin of the disorders, we ovariectomized (OVX) the mice and a week later analyzed LH and FSH levels. Over 10-fold higher LH and over 20-fold higher FSH confirmed the successful OVX (compare levels in **Figures 3**, **4**). Interestingly, LH remained significantly higher in OVX KO mice (8 ng/ml) compared to OVX WT mice (6 ng/ml), while there was no difference in FSH levels between WT and *Fmr1* KO females after OVX. Therefore, increased LH in unmodified animals likely stems from central dysregulation, while increased FSH involves ovaries.

### Increased innervation and vascularization in the ovaries

**Figure 5:**
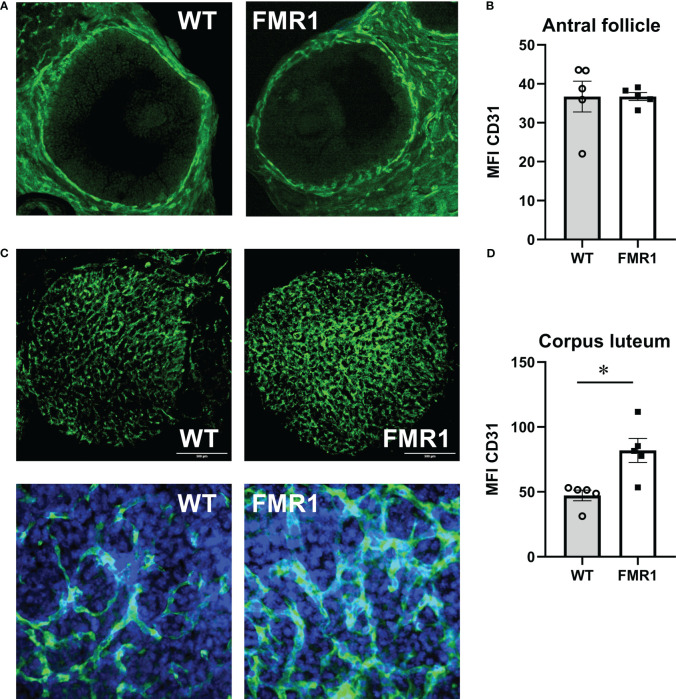
*Fmr1* KO have increased corpus luteum vascularization. Ovaries were sectioned and stained with antibodies to CD31 (PECAM-1, Platelet endothelial cell adhesion molecule) to visualize vascularity. Mean fluorescent intensity (MFI) was determined using Fiji imageJ. Consistent areas were used to quantify fluorescence intensity. **(A)** Representative images of antral follicles. **(B)** MFI quantification. **(C)** Corpora lutea representative images; top, 1.6 mm x 1.6 mm area, CD31 green; bottom, 300 μm x 300 μm area, CD31, green, DAPI, blue. **(D)** MFI quantification. Statistical significance, indicated with a * (p < 0.05) was determined with t-test followed by Tukey’s *post hoc* test.

To address seemingly discordant results, that *Fmr1* KO females exhibit higher levels of ovarian hormones than WT controls, which can provide negative feedback, and also increased FSH, which as revealed by ovariectomy is due to ovarian dysregulation, we analyzed ovarian vascularization and innervation. Using an endothelial cell marker, CD31 we stained ovarian sections and analyzed vascularization around follicles and corpora lutea. Follicles from WT and *Fmr1* KO had the same degree of vascularization (**Figure 5A**, representative image; **Figure 5B**, quantification). However, corpora lutea (CL) were more highly vascularized in *Fmr1* KO than in WT mice (**Figure 5C** low magnification, top). Higher magnification revealed more abundant and thicker vasculature in the *Fmr1* KO CL (**Figure 5C**, bottom, **Figure 5D**, quantification).

**Figure 6:**
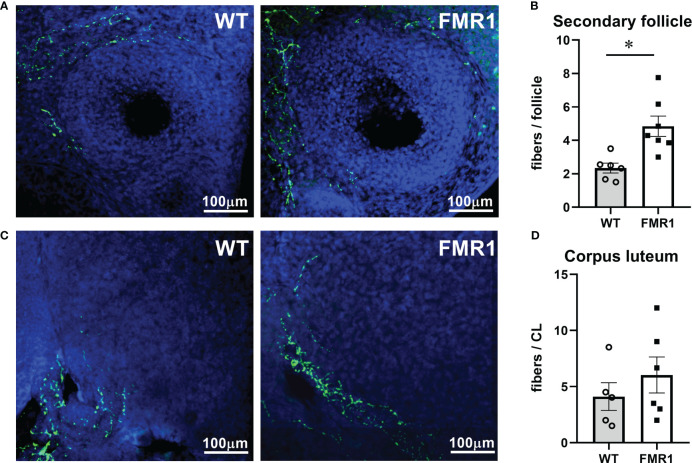
*Fmr1* KO have higher innervation of growing follicles. Ovaries were sectioned and stained with antibodies to tyrosine hydroxylase (TH, green) to visualize innervation around secondary follicles **(A)** and corpora lutea **(C)**. Fibers that surround the follicles and penetrate theca layer were counted **(B, D)**. * indicates significant difference.

Although FMRP is expressed at high level in neurons, ovarian innervation was not previously examined to possibly explain ovarian phenotype observed in *Fmr1* KO mice. Using antibodies to tyrosine hydroxylase, the rate-limiting enzyme in catecholamine synthesis, we counted numbers of neuronal fibers that reach the theca layer of growing follicles. Secondary follicles in *Fmr1* KO ovaries had significantly more neuronal fibers than WT follicles; 4.8 average fibers per secondary follicle in KO compared to 2.3 average fibers in WT (**Figure 6A**, representative images; **Figure 6B** quantification). Innervation of CLs was very variable within each animal and between animals, and we did not identify significant differences between WT and KO mice (**Figure 6C** bottom left quarter of CL with innervation presented, **Figure 6D**, quantification). Together, ovarian histology demonstrated increased vascularization of corpora lutea and increased innervation of secondary follicles in *Fmr1* KO mice.

### Altered levels of synaptic molecules in the hypothalamus of *Fmr1* KO mice

**Figure 7:**
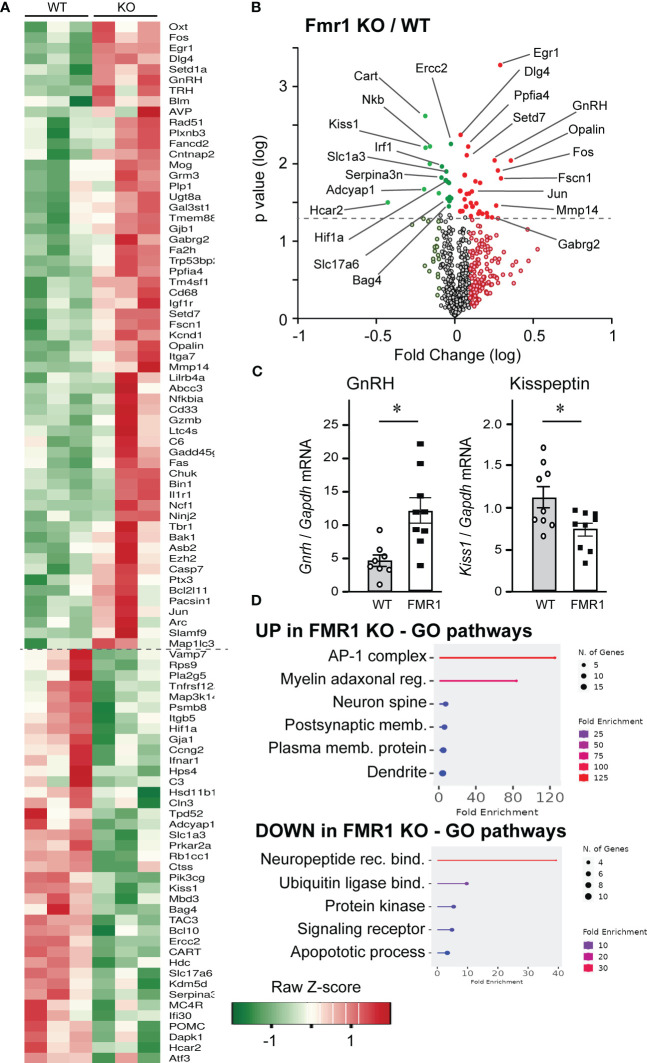
Nanostring analysis demonstrates significant changes in hypothalamic gene expression in *Fmr1* KO mice. **(A)** Hypothalami from 3 mice per group were dissected and 50 ng RNA used in Nanostring analysis. Heatmap indicates gene expression changes in *Fmr1* KO hypothalami that are <0.8-fold and >1.2-fold over WT. **(B)** Data were plotted as log fold change on x-axis *vs*. log p value on y-axis, and dashed line indicates significance. Red indicates genes that are increased in KO compared to WT mice, while green indicates genes that are decreased in KO compared to WT. Genes below the dotted line, light red and light green did not reach significance. **(C)** qPCR of the hypothalamus confirms changes in GnRH and kisspeptin expression. Each point represents one animal and bars represent group average. Statistical significance is indicated with *, determined by t-test followed by Tukey’s *post hoc* test. **(D)** Gene ontology pathway analysis indicates upregulated (top) and downregulated (bottom) pathways in the *Fmr1* KO mice.

Our results demonstrate elevated LH before and after ovariectomy, which is regulated by GnRH secretion. To address the mechanisms of changed LH, we examined changes in the hypothalamus, first focusing on gene expression changes caused by the loss of *Fmr1* gene. Although FMRP is an RNA-binding protein that regulates protein levels of its targets, genome-wide changes in the RNA expression signatures can identify pathogenic pathways that may be indirectly regulated by FMRP. Especially since FMRP regulates levels of several transcription factors and other transcriptional regulators, which may exhibit extensive changes on the transcriptome. Previous analyses of *Fmr1* KO transcriptomes focused on embryonic hippocampus and cortex, and identified overexpression of immune-related genes and downregulation of genes implicated in behavioral phenotype (78). For that reason, we used Nanostring neuroinflammation panel, which contains 770 genes implicated in neurological disorders, neuronal injury, neurotransmission, neuron-glia interactions, neuroplasticity, cell integrity, neuroinflammation, and metabolism; and added 30 custom probes for hypothalamic neuropeptides and their receptors. The complete list of genes that changed in KO compared to WT is presented by the heatmap (**Figure 7A**). There were 59 genes that were upregulated >120% from WT levels, and 39 genes that were downregulated <80% of WT levels, delineated with a dashed line. Significant changes in expression of neuropeptides and other genes of interest were highlighted in the volcano plot (**Figure 7B**, log fold change *vs*. log p-value, statistically significant change in expression indicated with a dashed line; red, upregulated genes in KO compared to WT; green, downregulated gene in KO compared to WT). Immediate early gene, transcription factors *Egr1*, *Fos* and *Jun*, that are used as markers of neuronal activation, were upregulated in *Fmr1* KO mice. Genes encoding GABA_A_ receptor γ2 subunit, (GABARγ2, *Gabrg*2) which is the obligatory subunit of the pentameric GABA_A_ receptor (79); and PSD-95 (*Dlg4*), a postsynaptic scaffolding protein anchoring glutamate receptors, were upregulated in KO mice. *Ppfia4*, involved in neurotransmitter release, and *Opalin*, important for oligodendrocyte differentiation, were also upregulated. On the other hand, genes correlated with DNA repair, *Ercc2*; neurodegenerative disorders, *Serpina3n*; hypoxia, *Hif1a*; and apoptosis, *Hcar2* and *Bag4*, were downregulated. Importantly, genes encoding GLAST, *Slc1a3*, and VGLUT2, *Slc17a6*, were also downregulated. Of interest, neuropeptide gene encoding GnRH, *Gnrh1*, was upregulated, while genes for kisspeptin, *Kiss1*, neurokinin B, Nkb, *Tac3*; and cocaine and amphetamine regulated transcript, *Cart*, were downregulated. We confirmed changes in *Gnrh1* and *Kiss1* expression by qPCR of hypothalamic lysates (**Figure 7C**). GO pathway analysis indicated that pathways such as AP1 complex, comprised of *Fos* and *Jun*, myelin adaxonal regulation, spine and dendrite development were upregulated, while neuropeptide binding, ubiquitin ligase binding, and receptor signaling pathways were downregulated (**Figure 7D**).

**Figure 8:**
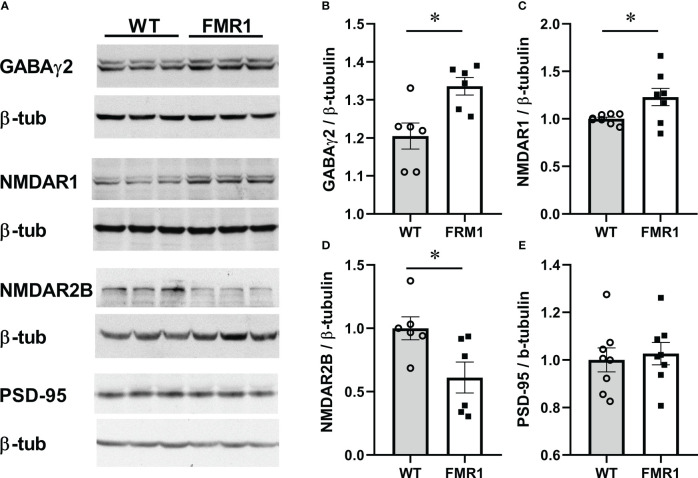
*Fmr1* KO females have higher levels of the obligatory GABA_A_ receptor subunit in hypothalami. Hypothalami were dissected and protein levels analyzed by western blotting. Representative blots are shown in **(A)**. Protein levels from 6 mice per group were quantified using Chemidoc and levels of neuronal proteins normalized to β-tubulin **(B–E)**. Statistical significance (p < 0.05), determined with t-test followed by Tukey’s *post hoc* test, is indicated with a *.

Considering changes in excitation/inhibition synaptic balance in the cortical neurons of *Fmr1* KO mice (80), we next investigated the levels of several synaptic proteins in hypothalamus at the protein level. Although previous studies showed changes in neurotransmitter receptor levels, such as GABA_A_ receptor for GABA, and NMDA receptor for glutamate, in several brain regions of male *Fmr1* KO mice, no studies were done in females or in the hypothalamus. Hypothalami were dissected from diestrus female brains, and levels of synaptic molecules were measured by western blotting. We analyzed GABARγ2 (Gabrg2) levels in the hypothalami of KO and control female mice in diestrus, since GABA transmission activates GnRH neurons, *via* activation of GABA_A_ receptor (26, 81), and determined that *Fmr1* KO females had significantly higher levels of GABARγ2 GABA_A_ receptor than controls (**Figure 8A**, representative western blots; **Figure 8B** quantification). Protein levels correlate with gene expression analysis, indicating that GABARγ2 may be indirectly regulated by Fmrp. We then analyzed levels of the glutamatergic NMDA receptors (NMDARs, or NRs) (82, 83), since 30-50% of GnRH neurons respond to NMDA (84, 85). NR1 is an obligatory subunit that forms a heterotetramer with either 2A or 2B subunits (other isoforms are less frequent). We determined that NR1 (Nmdar1, GluN1) levels were increased in the hypothalami of KO mice compared to controls (**Figure 8C**). Depending on the NR2 isoform, NMDAR is localized at synapses or extrasynaptically with different effects on long-term potentiation or negative feedback, respectively (64, 86). NR2B is localized extrasynaptically, and we determined that the levels of NR2B are lower in the KO mice compared to controls (**Figure 8D**). Genes encoding these proteins did not change at the transcriptional level, which indicates that they may be regulated by Fmrp at the protein level. We also analyzed levels of PSD-95, since PSD–95 anchors glutamate receptors (87), and determined that PSD-95 protein levels were the same in *Fmr1* KO and controls, which is contrary to gene expression studies and again points to direct regulation by Fmrp (**Figure 8E**). Taken together, an increase in the synaptic GABA_A_ and NR1 receptors, and a decrease in extrasynaptic NR2B, may contribute to altered activity of hypothalamic neurons in *Fmr1* KO female mice.

### Lack of Fmrp changes GnRH neuron connectivity

**Figure 9:**
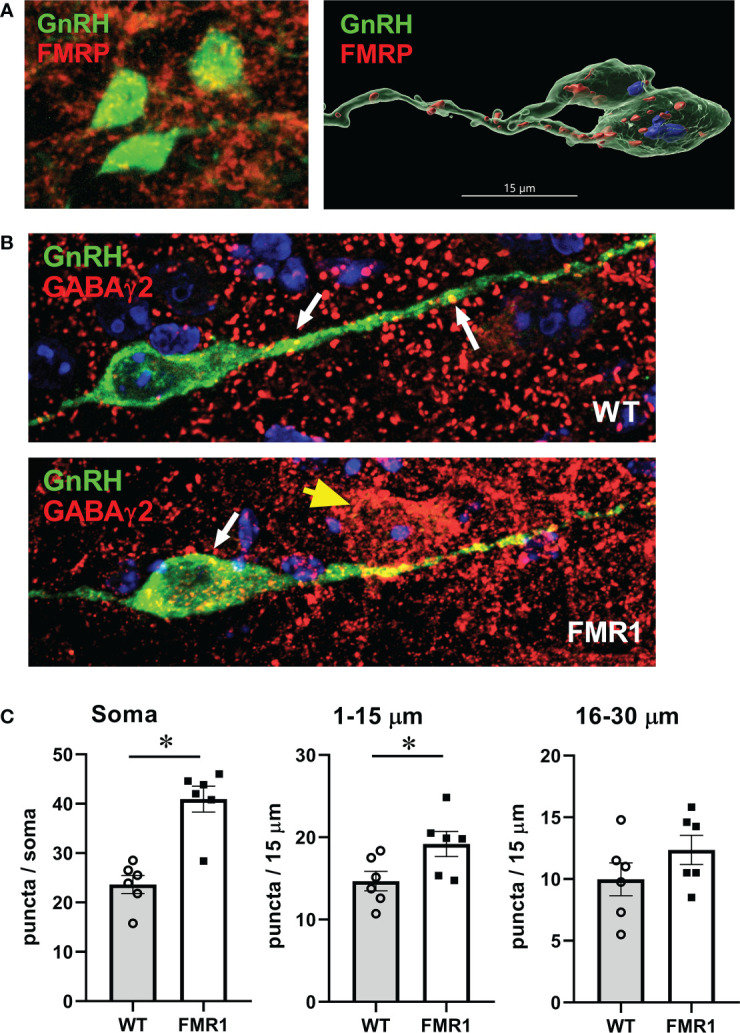
GnRH neurons of *Fmr1* KO females have more GABA_A_ receptors. **(A)** GnRH neurons (green) express Fmrp (red, top panels). **(B)** Representative GnRH neurons (green) from WT control (top panel) and *Fmr1* KO mice (bottom panel) co-stained with GABAγ2 antibody (red). **(C)** Quantification of **(B)** Each point represents one animals and an average of 15-20 neuron per animal, and bars represent group means. Panels represent counts in the whole soma, along the first 15 μm segment of the process (1-15 μm) and second 15 μm segment (16-30 μm) from the soma, as indicated above. Statistical significance (p < 0.05), determined with t–test followed by Tukey’s *post hoc* test, is indicated with a *.

To determine if Fmrp loss alters neurotransmitter receptor levels specifically in GnRH neurons, we first confirmed that 82% of GnRH neurons express Fmrp protein (**Figure 9A**, left image, confocal microscopy, right image, 3D reconstruction to demonstrate Fmrp inside GnRH neurons). Antibody specificity was determined by staining the hypothalami from *Fmr1* KO mice (**Supplemental Figure 2**). We also analyzed GnRH neuron number in *Fmr1* KO mice to compare to WT to ascertain if increased in GnRH neuron number contributes to higher LH. There was no difference in the number of GnRH neurons in WT and KO mice (**Supplemental Figure 2**).

Since GABA can regulate GnRH neuron activity (26, 81), and we determined elevated GABAγ2 subunit of the GABA_A_ receptor in the hypothalami of KO mice compared to controls by western blot, we analyzed if GABA_A_ receptor immunoreactivity is increased specifically in GnRH neurons. To determine GABA_A_ receptor distribution in GnRH neurons, we immunostained 100 μm coronal sections of the preoptic area of the hypothalamus for GABAγ2 and GnRH. After staining, sections were imaged with high-resolution confocal microscopy. GABAγ2 receptors showed puncta-like distribution in GnRH neurons. GABAγ2 receptor puncta colocalized with GnRH immunoreactivity were identified by closely apposed puncta when no black pixels were visible between two signals in optical slices, and counted blind to condition by scrolling through the series of captured z-stack images for each GnRH soma and along the process, at 15-μm intervals, for each GnRH neuron. At least 15 neurons were counted from each mouse, and the average for each mouse was calculated (represented by a dot in the **Figure 9C** with bars representing group average). We determined that GABAγ2 puncta numbers increased significantly in the GnRH neuron soma and in the first 15 μm of the process proximal to the soma (**Figure 9C** quantification, **Figure 9B**, representative images, top WT, bottom *Fmr1* KO). The increase in GABAergic inputs in this area is significant, since this region of the neuron is the region where action potentials are initiated (88), and it exhibits synaptic plasticity during development and in different hormonal milieu (89–91). Since GABA is excitatory for GnRH neurons (20, 26–28), the alteration of the receptor levels may enhance GnRH neuron responsiveness and neuropeptide secretion, which in turn would cause changes in gonadotropin levels.

**Figure 10:**
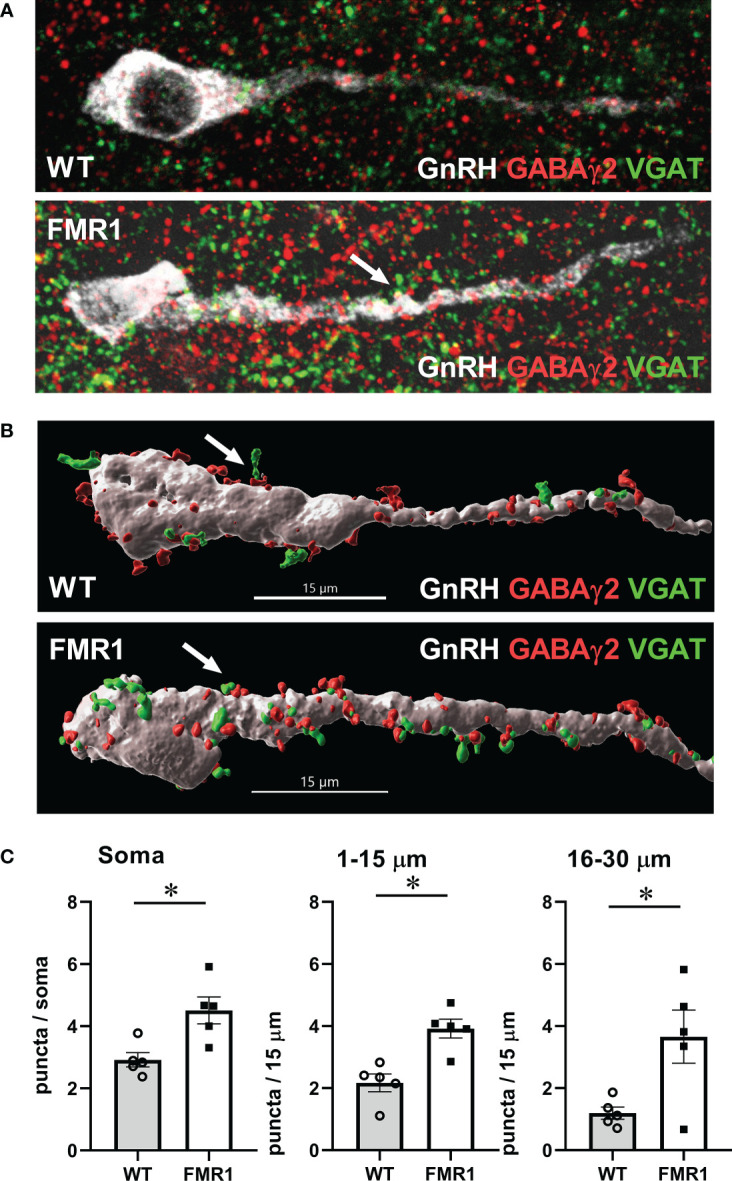
GnRH neurons of *Fmr1* KO females have more synaptic GABA_A_ receptors. **(A)** Representative images of triple stain for presynaptic VGAT (green), GABAγ2 receptor (red) and GnRH (white); **(B)** 3-D GnRH neuron models using Imaris software. VGAT (green) appositions to GABAγ2 receptor subunits (red) in GnRH neurons (white) were counted in 15-20 neurons per mouse, five mice from each group. **(C)** Quantification from different regions of the neuron, as above, is presented in panels, and significance indicated with *.

To determine GnRH innervation, we also analyzed appositions of GABAγ2 subunit of the GABA_A_ receptor with vesicular GABA transporter (VGAT), presynaptic marker of GABAergic terminals. We performed a triple stain for GnRH, GABAγ2 and VGAT, and as above, counted number of puncta where VGAT was in a close opposition to GABAγ2 in GnRH neurons (**Figure 10A**). We counted at least 15-20 neurons from each mouse, 5 pairs of mice; and used Imaris software to perform 3-D modeling of VGAT-GABA_A_ receptor appositions (**Figure 10B**). *Fmr1* KO mice had a higher number of GABAergic appositions in GnRH neuron soma and proximal process, in the segment 1–15 μm and segment 16–30 μm from the soma, than WT controls (**Figure 10C**). The increase in synaptic GABA_A_ receptors in the proximal process indicates higher innervation of GnRH neurons in the area that is plastic and receives synaptic input.

### Fmrp regulates LH pulsatility

**Figure 11:**
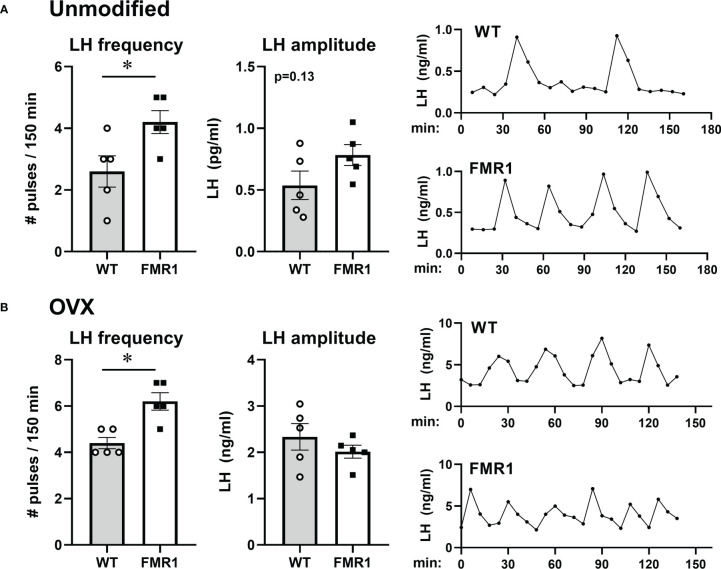
Higher LH/GnRH pulse frequency in *Fmr1* KO females before and after ovariectomy. LH pulse frequency reflects GnRH neuron activity. **(A)** Frequent tail-tip whole blood sampling over 3 hours demonstrate higher pulse frequency of LH in *Fmr1* KO female mice in diestrus. Representative profiles from WT control in the top and *Fmr1* KO at the bottom (right side), pulse frequency calculated from pulse profiles using DynPeak (left); amplitude was determined by subtracting the LH value at the peak from the basal value prior to the onset of the pulse and averaged for each mouse (middle). **(B)** LH pulse in ovariectomized (OVX) animals. Statistical significance (p < 0.05), determined with t-test followed by Tukey’s *post hoc* test, is indicated with a *.

Pulsatile secretion of LH strictly corresponds to GnRH secretion (49, 50). LH is used as an indicator of GnRH secretion, since GnRH secretion into median eminence cannot be measured in mice. To ascertain whether GnRH neuron secretion was affected, we measured LH pulses and used an ultrasensitive ELISA assay for LH (51) that allows for LH measurement in 5 µl of whole blood. Mice were acclimated for 2 weeks by daily tail massage. Serial sample collection every 8 min for 3 hours from the tail vein was performed (45, 52). Representative LH pulse profile from unmodified WT and KO mice are presented in **Figure 11A**, right side (WT control, top; *Fmr1* KO, bottom). Number of LH pulses per 2.5 hours of measurement were determined using DynPeak algorithm (53) and compared between genotypes. LH, and therefore GnRH, pulse frequency was significantly higher in *Fmr1* KO mice compared to WT controls (**Figure 11A**, left side). Amplitude was determined by subtracting the highest LH value from the basal value prior to the onset of the pulse and averaged for each mouse (**Figure 11A**, middle).

Pulsatile LH analyses were performed using ovariectomized animals as well. Frequency of LH secretion was faster in OVX *Fmr1* KO animals compared to OVX WT mice (**Figure 11B**, left; representative profiles right), while pulse amplitude was the same. Representative pulse profiles are shown on the right side. These experiments determined that the lack of Fmrp increased LH pulse frequency, indicating higher GnRH neuron activity corresponding to higher GABAergic innervation of GnRH neuron. It is possible the lack of Fmrp alters GnRH neuropeptide secretion leading to the faster GnRH pulse frequency and elevated LH, which contributes to ovarian dysregulation in young animals.

## Discussion

We sought out to uncover the effects of FMRP loss on hypothalamic GnRH neurons and ovarian function, which may help elucidate the mechanisms of early cessation of reproductive function in females with a mutation in the *FMR1* gene. Women with *FMR1* mutations comprise the majority of known genetic causes of premature ovarian failure (3, 35, 92). Premature ovarian failure or primary ovarian insufficiency is the most extreme manifestation of premature ovarian senescence and affects about 1% of women (34). Premature reproductive senescence affects approximately 10% of women and is characterized by an early depletion of ovarian follicles (34). Molecular causes of premature cessation of reproductive function in women with *FMR1* mutations and mechanisms underlying reproductive dysfunctions are still unknown. The hypothalamus was especially neglected in previous studies addressing *FMR1* function, or etiology of premature reproductive senescence. Our study is the first to examine the hypothalamic function of the *FMR1* gene. We analyzed mechanisms of reproductive disorders associated with *FMR1* mutations using the *Fmr1* KO female mice, since knockout mice lack Fmrp mimicking the loss of FMRP in humans with *FMR1* mutation. *Fmr1* KO female mice are useful model to study reproductive disorders in women with a mutation in the *FMR1* gene, as they exhibit early cessation of reproductive function similar to women with *FMR1* mutations. The main findings of this study implicate central mechanisms and ovarian innervation in reproductive disorders associated with the FMRP loss. We demonstrate that *Fmr1* KO female mice show higher GnRH neuron and ovarian follicle innervation, increased GnRH neuron secretion, and augmented gonadotropin levels, all of which may contribute to increased recruitment of ovarian follicles to the growing pool, corresponding to a higher number of corpora lutea and larger litters in young animals. These growing follicles exhibit increased innervation, which is associated with higher steroidogenesis. Together, our results point to hypothalamic mechanisms, specifically GnRH neuron connectivity, and ovarian innervation in the reproductive disorders associated with FMRP loss that have not been considered before.

Women with early menopause face not only infertility, but an increased risk of heart disease and osteoporosis (93–97). Most women are only diagnosed after their ovarian function has ceased, since they seek care due to infertility or amenorrhea. In that case, it is difficult to predict earlier hormonal changes, when ovarian reserves are relatively normal. As of yet, there are no screening strategies to detect women with increased risk before they are symptomatic (98). Most studies analyzing a role of FMRP have focused on males, while females are rarely included. Furthermore, cortical mechanisms attracted the attention of the investigators, because FXS is the most common monogenic cause of intellectual disability and autism. However, the mechanism underlying the dysregulation of reproductive function in *FMR1* mutations was not extensively studied. Several reports demonstrate elevated FSH in women affected with *FMR1* mutations showing early menopause (40, 41), which are consistent with our observations in mice. Since the primary reproductive defect in females with *FMR1* mutation is premature ovarian failure, gonadal origin was proposed. We demonstrate that primordial follicle number is unaffected by *Fmr1* loss, which agrees with previous studies that showed that women with *FMR1* mutation and mouse models of premutation have normal pool of primordial follicles (31, 38, 39, 70). This indicates that early ovarian development is not adversely affected by the loss of FMRP. Our results using complete KO model show larger litters and more corpora lutea, while premutation mouse models have smaller litters (31, 38), but reasons for differences are not clear. Our results do not preclude other intra-ovarian defects that may contribute to the early loss of follicles and early depletion, such as increased atresia as suggested in (38). Given that there is no difference in primordial follicle development, inappropriate ovarian response to gonadotropin stimulation, compounded by changes in gonadotropin levels, likely contributes to early cessation of reproductive function in *FMR1* mutations.

Removal of ovaries demonstrated that increased FSH depends on ovarian feedback. However, the increase in FSH is not a result of the lack of negative feedback as we show that in females, ovarian hormones, inhibin or steroid hormones, are higher than in controls, indicating that hormonal feedback to the pituitary and hypothalamus was present. Studies in women with *FMR1* mutations are inconclusive, with one study reporting unchanged inhibin (6), while the other found decreased inhibin (40). The latter study is the only one, to our knowledge, that analyzed steroid hormone levels and found decreased progesterone (40). The discrepancy may arise due to the age of the subjects, as discussed above. Increase in inhibin B levels in our results, implies that higher FSH occurs irrespective of inhibin feedback, in fact, may lead to increased inhibin, and thus, higher FSH may be the cause rather than the consequence of reproductive disorders.

Since ovarian hormones did not provide adequate explanation, we examined ovarian innervation and vascularization, and detected increased vascularization of corpora lutea which may correlate with higher progesterone levels (99). Increased progesterone may lead to higher FSH (100, 101). Fluorescent studies with endothelial cells marker reveal that large follicle vascularization is not changed, but corpora lutea exhibit increased vascularization. Developing corpus luteum is a site of rapid angiogenesis, under the influence of the vascular endothelial growth factor (VEGF) (99, 102, 103). Studies in several species determined that angiogenesis and VEGF induction is stimulated by LH (104–106). Thus, increased LH in our study likely contributes to increased vascularization of the corpus luteum. Treatment with VEGF antagonist demonstrated decreased progesterone (107). Therefore, increased progesterone in *Fmr1* KO mice we report here may be due to increased vascularization of corpora lutea. Several studies determined that progesterone could increase FSH at the transcriptional level, which is thought to be important for the specific secondary rise of FSH during luteal phase. Progesterone treatment in combination with estrogen, increased FSH, while antiprogestins blocked FSH secretion and mRNA expression during the preovulatory surge (108) and during the secondary rise (109). Therefore, our studies postulate that increased LH may cause increased angiogenesis during luteinization, which leads to higher progesterone, which in turn increases FSH, and together, may explain why increased LH is of hypothalamic origin, while increased FSH requires ovaries.

Ovaries receive sympathetic innervation *via* two routes (110–112). The superior ovarian nerve projections innervate the secretory component of the ovary. The fibers surround the developing follicles, but do not penetrate the granulosa layer or the corpus luteum. We determined that innervation of the follicles with fibers that originate from the superior ovarian nerve is increased. This innervation is required for steroidogenesis, since transection of the nerve reduced steroid hormone levels (113–115). Thus, higher steroid hormone levels may be a result of increased innervation. As discussed above, this may contribute to higher FSH levels in KO animals.

Increased LH likely stems from higher GnRH secretion, *via* increased GnRH pulse frequency, since GnRH from the hypothalamus strictly regulates LH secretion. We determined that GnRH neurons have increased GABAergic innervation, which is excitatory for GnRH neurons (20, 26–28). Therefore, it is possible that increased GABA tone leads to altered responsiveness of GnRH neurons to the pulse generator and the upstream regulatory network. Alternatively, constantly increased GABA input may lead to increased activity of GnRH neurons. The idea of enhanced activation of GnRH neurons is also supported by the observed increases in neurotransmitter receptor levels in the hypothalamus and specifically GnRH neurons. FMRP binds mRNAs that encode synaptic proteins (9–11) and previous studies reported altered levels of GABA_A_ receptors and NMDA receptors in several brain areas. Here, we further determined that GABA_A_ receptor abundance changes at the transcriptional level, while NMDARs change on the protein level, which may elucidate direct versus indirect regulation by FMRP. Contrary to the studies in the cortex and hippocampus, which detected decreased GABA_A_ receptor, we determined that GABA_A_ receptor levels are increased in the hypothalamus at the mRNA and protein levels. 30%-50% of GnRH neurons respond to NMDA (84, 85), and our studies in the hypothalamus agree with previous studies in the cortex that demonstrate increased NMDA receptors in *Fmr1* KO mice. Therefore, the increase in NMDA and GABA receptors, that are both excitatory for GnRH neurons, likely changes GnRH neuron function and GnRH neuropeptide secretion.

Therefore, both the hypothalamus and ovaries contribute to endocrine disruption that may lead to larger litters in young animals. Hypothalamic contribution to the etiology of early menopause has been underappreciated. We propose that changes in GnRH neuron and ovarian innervation contribute to changes in gonadotropin hormones, LH and FSH, levels. This may cause early depletion of ovarian follicles and premature cessation of reproductive function, which will be addressed in future studies. Together, our results point to hypothalamic mechanisms and ovarian innervation in the reproductive function disorders associated with FMRP loss that have not been considered before.

## Data availability statement

The data presented in the study are deposited in the Gene Expression Omnibus GEO repository, accession number GSE222723. link: https://www.ncbi.nlm.nih.gov/geo/query/acc.cgi?acc=GSE222723 these are fluorescent images and the background is meant to be black to demonstrate specific staining of the area of interest.

## Ethics statement

The animal study was reviewed and approved by UCR IACUC.

## Author contributions

PV and NL performed most of the experiment reported herein and data analyses. CJ determined reproductive phenotype. SB performed analyses of ovarian vasculature. IE provided valuable insight in Fragile X Syndrome pathology. DC conceived and guided the study and wrote the manuscript. PV: Conceptualization, Investigation, Formal Analysis, Visualization, Writing – Review and editing. NL: Investigation, Formal Analysis, Visualization, Writing – review and editing. CJ: Investigation, Formal Analysis, Visualization, Writing – review and editing. SB: Investigation, Formal Analysis, Visualization, Writing – review and editing. IE: Resources, Validation, Supervision, Writing – review and editing. DC: ORCid: 0000-0003-0692-1612 Conceptualization, Formal Analysis, Funding Acquisition, Project Administration, Supervision, Visualization, Writing – original draft and preparation. All authors contributed to the article and approved the submitted version.

## Supplementary material

Supplementary Figure 1
*Fmr1* KO females have higher *Lhb* and *Fshb* mRNA expression. Pituitary mRNA was analyzed by RT-qPCR to determine the expression of pituitary hormones. *Lhb* and *Fshb* are increased, while expression of other hormones is unchanged. Each point represents one animal, while bars represent group means. mRNA levels were normalized to B2M mRNA. Statistical significance (p < 0.05), determined with t-test followed by Tukey’s *post hoc* test, is indicated with a *.

Supplementary Figure 2 Left, specificity of antibody to FMRP is determined by staining hypothalamic sections from *Fmr1* KO mice. Right, every 5^th^ coronal section containing the preoptic area of the hypothalami from WT and *Fmr1* KO female mice were stained with anti-GnRH antibodies and GnRH neurons counted. Each point represents average number of neurons from all counted sections for one animal, while bars represent group means.
